# Control of Time Delay Force Feedback Teleoperation System With Finite Time Convergence

**DOI:** 10.3389/fnbot.2022.877069

**Published:** 2022-05-06

**Authors:** Jingwen Wang, Jiawei Tian, Xia Zhang, Bo Yang, Shan Liu, Lirong Yin, Wenfeng Zheng

**Affiliations:** ^1^School of Automation, University of Electronic Science and Technology of China, Chengdu, China; ^2^Department of Geography and Anthropology, Louisiana State University, Baton Rouge, LA, United States

**Keywords:** the teleoperation system, the terminal sliding mode control method, the neural network adaptive control method, the Lyapunov theory, tracking error

## Abstract

In order to make the teleoperation system more practical, it is necessary to effectively control the tracking error convergence time of the teleoperation system. By combining the terminal sliding mode control method with the neural network adaptive control method, a bilateral continuous finite time adaptive terminal sliding mode control method is designed for the combined teleoperation system. The Lyapunov theory is used to analyze the stability of the closed-loop system, and the position tracking error is able to effectively converge in time. Finally, the effectiveness of the proposed control scheme is verified by MATLAB Simulink numerical simulation, and the numerical analysis of the results shows that the method has better system performance. Compared with the traditional two-sided control method (TPDC) of PD time-delay teleoperation system, the control method in this paper has good performance, improves stability, and makes steady-state errors smaller and better tracking.

## Introduction

By improving the mechanical design of the teleoperation robot, as well as the control structure and algorithm of the system, the performance and application range of the teleoperation system have been greatly improved. The general remote operation robot system mainly includes the master module, operator module, master controller, communication channel, slave controller, slave environment, and so on. The general remote operation robot system has been applied in many fields, such as unmanned submersible (Sayers and Paul, 1994), space robots (Bejczy, 1994; Wright et al., 2006), remote surgery robots (Sayers and Paul, 1994; Tang et al., 2020a), teleoperation robots (DiMaio et al., 2011), etc.

From the research status of teleoperation system, for the uncertain control system, the control algorithm based on the sliding mode can achieve well-control, and it is robust to the internal parameter uncertainty and external interference, which has been widely used (Feng et al., 2002; Yu et al., 2005; Li and Huang, 2010; Neila and Tarak, 2011; Nekoukar and Erfanian, 2011). But in the above literature, the sliding mode control method is linear. The state variables of the system with linear sliding mode control strategy converge to the equilibrium point on the sliding surface at an exponential rate. Although the appropriate parameters can be adjusted arbitrarily and quickly, the power system cannot reach stable in a limited time.

In the practical application of teleoperation system, it is more desirable to complete the error convergence in finite time, because it can complete the task better and faster. In order to obtain the characteristic that the tracking error of the system converges to zero in finite time, St (Yu and Man, 2002) proposed a terminal sliding mode control method, using non-linear sliding mode hyperplane for the first time. Then, many studies have carried out in-depth research and improvement on this method (Salcudean et al., 2000; Xu and Yao, 2001; Nuno et al., 2008; Zhang et al., 2009; Nekoukar and Erfanian, 2011; Liu and Zhang, 2013). Compared with the control method based on the linear sliding mode hyperplane, the terminal sliding mode control method has better characteristics, such as faster, finite time convergence and so on. However, in practical engineering, it is not only difficult to realize the existing terminal sliding mode controller, but also, when the design parameters are not suitable, there will be a singular problem (Guo et al., 2021; Ma et al., 2021; Zhang et al., 2021). In order to solve these problems, there are many control methods. However, for the design of the remote operation system controller, these methods are not applicable. In teleoperation system, not only the influence of the operator module and the environment module but also the time delay of the communication channel should be considered. Therefore, the finite time sliding mode control strategy for a robot cannot be directly used in bilateral teleoperation system (Tang et al., 2020a). So, we need to further study the sliding mode control strategy of teleoperation system and propose a new algorithm to obtain the appropriate switching function and controller so as to ensure the asymptotic stability of the sliding mode in the motion process of the system, and then complete the finite time tracking error convergence and improve the overall stability and tracking performance of the system.

Therefore, in this paper, in order to make the teleoperation system with time-delay force feedback more practical, a finite time non-linear terminal sliding mode adaptive bilateral control method is designed for the teleoperation system with constant time delay. Meanwhile, the constant time delay generated by the communication channel in the teleoperation system and the influence of uncertainties on the model are solved, and the tracking error of the teleoperation system can converge in finite time (Li et al., 2015, 2016).

## Methods

The main goal of this paper is to design a two-sided controller based on the position error control structure, considering the internal friction, external interference, and constant time delay between the master robot and the slave robot in the teleoperation system to make the convergence time of the position tracking error of the system converge to 0 in a finite time. Similarly, the RBF neural network adaptive method is also used to approximate the uncertainty of the system model, but the treatment of the uncertainty is different (Liu et al., 2018; Dankwa and Zheng, 2019; Yang et al., 2019; Xu et al., 2020).

### Controller Design and Stability Analysis of Teleoperation System

In the control of teleoperation system with forward channel delay and reverse channel communication delay, considering the mechanical internal friction and external interference of the master robot and the slave robot in the system, our control goal is to calculate the control torque input of the master robot and the slave robot, respectively, so that the position error between the master robot and the slave robot in the teleoperation system can converge to 0 in finite time and guarantee the stability of the system (Li et al., 2017a,b, 2020; Zheng et al., 2017; Yin et al., 2019; Chen et al., 2020; Tang et al., 2020b).

**Figure 1:**
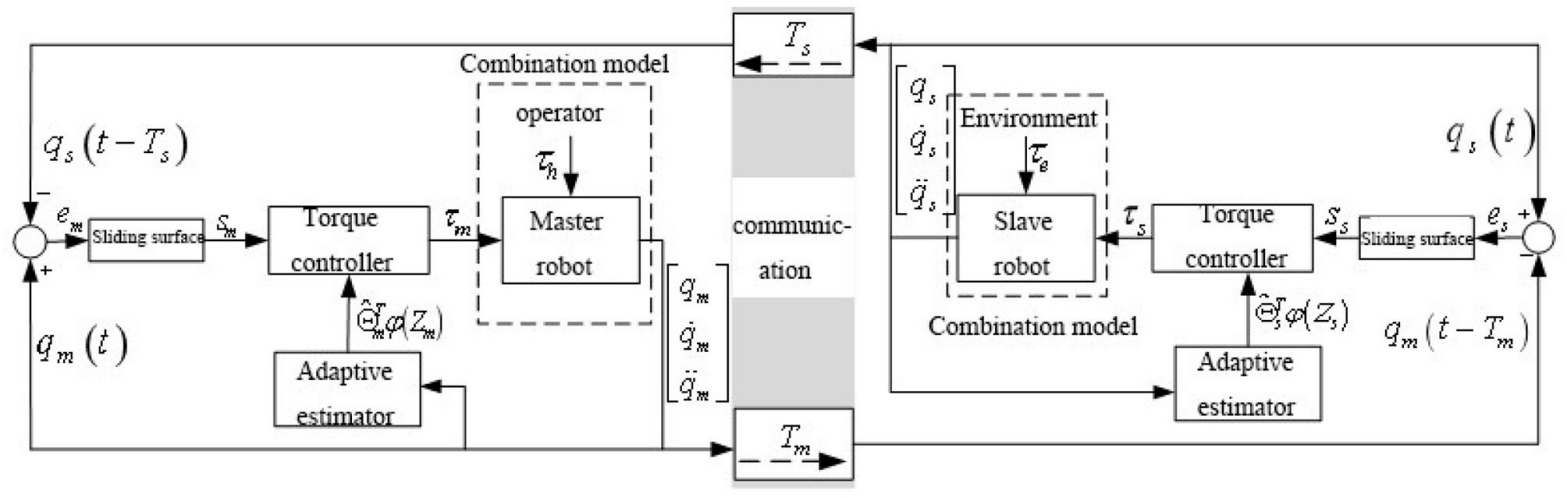
A control structure diagram of time delay force feedback teleoperation system with finite time convergence.

In this paper, the control block diagram of time-delay force feedback teleoperation system based on position error structure with finite time convergence is shown in Figure 1. Considering the influence of the constant time delay and the non-linear uncertainties of the system model on the teleoperation system, as well as the singularity and chattering problems of the sliding mode control, a finite time non-linear sliding mode adaptive bilateral controller is adopted. Compared with the linear sliding mode controller, the controller can make the teleoperation system work well. The tracking error of the system can converge to 0 quickly and finitely, and ultimately ensure the global stability of the teleoperation system.

#### Controller Design

From the control block diagram of time-delay force feedback teleoperation system based on position error structure shown in Figure 1, it can be defined that the position tracking error of the master robot and the slave robot is as the following Formula (1):


(1) $$\begin{array}{l} e_{m}\;=\;q_{m}\;-\;q_{s}\left(t\;-\;T_{m}\right),\;e_{s}\;=\;q_{s}\;-\;q_{m}\left(t\;-\;T_{s}\right) \end{array}$$

Here, *T*_*m*_is the communication delay of the forward channel, and *T*_*s*_ is the communication delay of the reverse channel. The position and velocity errors of the master robot and the slave robot are defined as the following Formula (2):


(2) $$\begin{array}{l} {ė}_{m}\;=\;{\overset{∙}{q}}_{m}\;-\;{\overset{∙}{q}}_{s}\left(t\;-\;T_{s}\right),\;{ė}_{s}\;=\;{\overset{∙}{q}}_{s}\;-\;{\overset{∙}{q}}_{m}\left(t\;-\;T_{m}\right) \end{array}$$

Then, based on the non-singular terminal sliding mode method, the sliding mode function is defined as follows:


(3) $$\begin{array}{l} s_{m}\;=\;e_{m}\;+\;\alpha_{m}sig{\left(e_{m}\right)}^{\varepsilon_{m}}\;+\;\beta_{m}sig{\left({ė}_{m}\right)}^{\gamma_{m}} \end{array}$$

(4) $$\begin{array}{l} s_{s}\;=\;e_{s}\;+\;\alpha_{s}sig{\left(e_{s}\right)}^{\varepsilon_{s}}\;+\;\beta_{s}sig{\left({ė}_{s}\right)}^{\gamma_{s}} \end{array}$$

Where, $sig{\left(\xi \right)}^{\alpha }={\left[|\xi_{1}{|}^{\alpha_{1}}sign(\xi_{1}),\;|\xi_{2}{|}^{\alpha_{2}}sign(\xi_{2}),\;\cdots ,|\xi_{n}{|}^{\alpha_{n}}sign(\xi_{n})\right]}^{T},\;\xi \;=\;{\left[\xi_{1},\xi_{2},\;\cdots ,\xi_{n}\right]}^{T}\in R^{n},\alpha_{1},\alpha_{2},\cdots ,\alpha_{n}\;>\;0.\;s_{i}\;=\;[s_{i_{1}},\;s_{i_{2}},\;\cdots ,s_{n_{1}}]\;\in \;{ℜ}^{n},\alpha_{i}\;=\;diag(\alpha_{i1},\alpha_{i2},\cdots ,\alpha_{in})\text{ and }\beta_{i}\;=\;diag(\beta_{i1},\beta_{i2},\cdots ,\beta_{in})$ are positive diagonal matrices, and ε_*i*_*j*__ > γ_*i*_*j*__, 1 < γ_*i*_1__, γ_*i*_1__, ⋯ , γ_*i*_*n*__ <2; *i* = *m, s*; *j* = 1, 2, ……, *n*.


(5) $$\begin{array}{l} S\;=\;e\;+\;\alpha \;sig{\left(e\right)}^{\varepsilon }\;+\;\beta \;sig{\left(ė\right)}^{\gamma } \end{array}$$

Through a Formula (5), the derivation of the Formula (3) and the Equation (4) is obtained


(6) $$\begin{array}{l} {ṡ}_{m}\;=\;{ė}_{m}\;+\;\varepsilon_{m}\alpha_{m}diag\left({\left|e_{m}\right|}^{\varepsilon_{m}\;-\;1}\right){ė}_{m}\\ \;+\;\gamma_{m}\beta_{m}diag\left({\left|{ė}_{m}\right|}^{\gamma_{m}\;-\;1}\right){ë}_{m} \end{array}$$

(7) $$\begin{array}{l} {ṡ}_{s}\;=\;{ė}_{s}\;+\;\varepsilon_{s}\alpha_{s}\;\text{diag }\left({\left|e_{s}\right|}^{\varepsilon_{s}\;-\;1}\right){ė}_{s}\;+\;\gamma_{s}\beta_{s}\;\text{diag}\\ \;\left({\left|{ė}_{s}\right|}^{\gamma_{s}\;-\;1}\right){ë}_{s} \end{array}$$

In order to solve the influence of system model uncertainty$,\;P_{i}\left(q_{i},{\overset{∙}{q}}_{i},{\ddot{q}}_{i}\right)$on system stability, this paper uses radial basis function neural network to approximate it. As a result:


(8) $$\begin{array}{l} P_{i}\left(q_{i},{\overset{∙}{q}}_{i},{\ddot{q}}_{i}\right)\;=\;\Theta_{i}^{T}\varphi \left(Z_{i}\right)\;+\;\delta_{i}\left(Z_{i}\right) \end{array}$$

According to the expression of the uncertainty$,P_{i}\left(q_{i},{\overset{∙}{q}}_{i},{\ddot{q}}_{i}\right)\;$of the system model, we can choose the input signal $Z_{i}=\left[q_{i}^{T},{\overset{∙}{q}}_{i}^{T},\;{\ddot{q}}_{i}^{T}\right]$ of the network, δ_*i*_(*Z*_*i*_) as the bounded estimation error, which satisfies ∥δ_*i*_(*Z*_*i*_)∥ ≤ ε_*i*_, ε_*i*_ is a constant. Θ_*i*_ is the weight that needs to be adjusted.

The terminal sliding mode control method and the radial basis function estimation method are used to design appropriate controllers for the master robot and the slave robot in the teleoperation system with constant time delay.


(9) $$\begin{array}{l} \tau_{m}\;=\;-M_{om}\left(q_{m}\right)\left(I\;+\;F_{m}\right)\beta_{m}^{-1}\gamma_{m}^{-1}sig{\left({ė}_{m}\right)}^{2\;-\;\gamma_{m}}+M_{om}\\ \left(q_{m}\right){\ddot{q}}_{s}\left(t-T_{s}\right)\;+\;C_{om}\left(q_{m},{\overset{∙}{q}}_{m}\right){\overset{∙}{q}}_{m}\left(t\right)+G_{om}\left(q_{m}\right)\\ \;-M_{om}\left(q_{m}\right)\left(K_{m}s_{m}+B_{m}sig{\left(s_{m}\right)}^{\rho_{m}}\right)\\ \;-\;\frac{s_{m}}{∥s_{m}∥}{\tilde{\Theta }}_{m}\varphi \left(Z_{m}\right)-\frac{{\left(h_{m}\right)}^{T}}{∥h_{m}∥}{\hat{\Theta }}_{m}\varphi \left(Z_{m}\right) \end{array}$$

(10) $$\begin{array}{l} \tau_{s}=-M_{os}\left(q_{s}\right)\left(I+F_{s}\right)\beta_{s}^{-1}\gamma_{s}^{-1}sig{\left({ė}_{s}\right)}^{2-\gamma_{s}}+M_{os}\left(q_{s}\right){\ddot{q}}_{m}\left(t-T_{m}\right)\\ \;+C_{os}\left(q_{s},{\overset{∙}{q}}_{s}\right){\overset{∙}{q}}_{s}\left(t\right)+G_{os}\left(q_{s}\right)-M_{os}\left(q_{s}\right)\left(K_{s}s_{s}+B_{s}sig{\left(s_{s}\right)}^{\rho_{s}}\right)\\ \;-\frac{s_{S}}{∥s_{S}∥}{\overset{∙}{\sim }\Theta }_{S}\varphi \left(Z_{S}\right)-\frac{{\left(h_{S}\right)}^{T}}{∥h_{S}∥}{\overset{∙}{\Theta }}_{S}\varphi \left(Z_{S}\right) \end{array}$$

Here, for all the *K*_*i*_, *B*_*i*_ are positive diagonal matrices, where *i* = *m, s*, and $0\;<\;\rho_{i}\;<\;1,{\tilde{\Theta }}_{i}\;=\;\Theta_{i}\;-\;{\hat{\Theta }}_{i},h_{i}\;=\;s_{i}^{T}\gamma_{i}\beta_{i}diag\left({\left|{ė}_{i}\right|}^{\gamma_{i}\;-\;1}\right)M_{oi}^{-1}\left(q_{i}\right),F_{i}\;=\;\alpha_{i}\varepsilon_{i}diag\left({\left|e_{i}\right|}^{\varepsilon_{i}\;-\;1}\right),{\hat{\Theta }}_{i}$ is the estimated value of Θ_*i*_, and the estimation law adopted is as the following Formula (11):


(11) $$\begin{array}{l} {\hat{\Theta }}_{i}=\Lambda_{i1}\varphi \left(Z_{i}\right){\overset{∙}{q}}_{i}^{T}-\Lambda_{i1}\left({\hat{\Theta }}_{i}-\Theta_{i}^{*}\right) \end{array}$$

Here, Λ_*i*1_, Λ_*i*2_ are the normal number; $\Theta_{i}^{*}$ is the nominal value of Θ_*i*_, *i* = *m, s*.

#### Analysis of System Stability and Tracking Performance

The time-delay force feedback teleoperation system includes a bilateral position control closed loop, and its control structure is shown in Figure 1. The stability of the closed-loop teleoperation system and the position tracking performance analysis of bilateral position control are discussed below.

Theorem 5: In the case of constant forward and reverse channel delays, uncertain model parameters, and external interference, the non-linear sliding surface of Formulas (3) and (4) is selected, and the bilateral continuous terminal sliding mode control with effective time convergence of Formulas (9) and (10) is adopted. The controller and the control of neural network adaptive law described in the Formula (11) are as follows:

(1) The whole closed-loop system is globally stable, and all closed-loop signals are globally bounded.
(2) In the whole closed-loop teleoperation system, the tracking error of the master robot and the slave robot can converge to 0 in finite time.

Prove (1): now, the Lyapunov candidate functions can be constructed as the following Formula (12)


(12) $$\begin{array}{l} V\;=\;V_{1}\;+\;V_{2} \end{array}$$

Among them, $V_{1}\;=\;\underset{j\;=\;m,s}{\sum }\;\frac{1}{2}S_{j}^{T}S_{j}$, $V_{2}\;=\;\frac{1}{2}\underset{i=m,\;s}{\sum }Tr\left({\tilde{\Theta }}_{i}^{T}\Lambda_{i1}^{-1}{\tilde{\Theta }}_{i}\right)$. The derivative of *V*_1_ is obtained as Formula (13):


(13) $$\begin{array}{l} {\overset{∙}{V}}_{1}\;=\;\underset{j\;=\;m,s}{\sum }s_{j}^{T}{Ṡ}_{j} \end{array}$$

By substituting Formula (6) and Formula (7) into Formula (13), the results are as Formula (14):


(14) $$\begin{array}{c} V_{1}=\sum_{i=m,s}\{-s_{i}^{T}\bar{K_{i}}s_{i}-s_{i}^{T}\bar{B_{i}}sig{\left(s_{i}\right)}^{\rho_{i}}+s_{i}^{T}\gamma_{i}\beta_{i}\text{ diag}\\ {\left|e_{i}\right|}^{\gamma_{i}-1})M_{oi}^{-1}(q_{i})\times (P_{i}+\frac{h_{i}^{T}}{∥h_{i}∥}{\hat{\Theta }}_{i}^{T}\varphi (Z_{i}))-s_{i}^{T}\gamma_{i}\beta_{i}\text{ diag}\\ \left({\left|e_{i}\right|}^{\gamma_{i}-1})M_{oi}^{-1}(q_{j})\frac{s_{i}}{∥s_{i}∥}{\tilde{\Theta }}_{i}^{T}\varphi (Z_{i}\right) \end{array}$$

Here$,\;S=\left[s_{m}^{T},s_{s}^{T}\right]$,${\text{Ψ}}_{1}\;=\;diag\left(\bar{K_{m}},\bar{K_{s}}\right)$, ${\text{Ψ}}_{2}=diag\left(\bar{B_{m}},\bar{B_{s}}\right)$, ${\bar{\psi }}_{1}$and ${\bar{\psi }}_{2}$ are eigenvalues of Ψ_1_ and Ψ_2_. Among which, $\bar{K_{i}}=\gamma_{i}\beta_{i}diag\left({\left|{ė}_{i}\right|}^{\gamma_{i}\;-\;1}\right)K_{i}\;\in \;R^{n\times n}$, $\bar{B_{i}}=\gamma_{i}\beta_{i}diag\left({\left|{ė}_{i}\right|}^{\gamma_{i}\;-\;1}\right)B_{i}\in R^{n\times n}$.

According to the definition above ~∙Θi = -^∙Θi, and ${\overset{∙}{\sim }\;\Theta }_{i}\;=\;\Theta_{i}\;-\;\Theta_{i}^{*}$. We can get the following result by deriving from *V*_2_.


(15) $$\begin{array}{l} {\overset{∙}{}V}_{2}\;=\;-\underset{i=m,s}{\sum }\left(Tr\left({\tilde{\Theta }}_{i}^{T}\varphi \left(Z_{i}\right)\right){\overset{∙}{q}}_{i}^{T}-\frac{\Lambda_{i2}}{\Lambda_{i1}}{\tilde{\Theta }}_{i}^{T}\left({\tilde{\Theta }}_{i}-{\bar{\Theta }}_{i}\right)\right) \end{array}$$

Because $∥s_{j}^{T}\gamma_{j}\beta_{j}\;\text{diag}\;\left(|{ė}_{j}{|}^{\gamma_{j}-1}\right)M_{oj}^{-1}\left(q_{j}\right)P_{j}∥\;\leq \;∥s_{j}^{T}\gamma_{j}\beta_{j}\;\text{diag}\;\left(|{ė}_{j}{|}^{\gamma_{j}-1}\right)M_{oj}^{-1}\left(q_{j}\right)∥∥\;P_{j}∥,\;\text{and}\;{\tilde{\theta }}_{i}^{T}\left({\tilde{\theta }}_{i}-{\bar{\theta }}_{i}\right)\leq \frac{1}{2}||{\tilde{\theta }}_{i}|{|}_{F}^{2}\;-\;\frac{1}{2}||{\bar{\theta }}_{i}|{|}_{F}^{2}$, there are


(16) $$\begin{array}{c} \dot{V}\leq \sum_{i=m,s}\left\{-s_{i}^{T}\bar{K_{i}}s_{i}-s_{i}^{T}\bar{B_{i}}sig{\left(s_{i}\right)}^{\rho_{i}}-s_{i}^{T}\gamma_{i}\beta_{i}diag(|{\dot{e}}_{i}{|}^{\gamma_{i}-1}\right)\\ M_{oi}^{-1}(q_{i})\frac{s_{i}}{∥s_{i}∥}{\tilde{\Theta }}_{i}^{T}\varphi (Z_{i})\}+\\ \sum_{i=m,s}\left(-Tr({\tilde{\Theta }}_{i}^{T}\varphi (Z_{i})){\dot{q}}_{i}^{T}-\frac{\Lambda_{i2}}{2\Lambda_{i1}}∥{\tilde{\Theta }}_{i}{∥}_{F}^{2}+\frac{\Lambda_{i2}}{2\Lambda_{i1}}∥{\bar{\Theta }}_{i}{∥}_{F}^{2}\right) \end{array}$$

Therefore, *V*(*t*)≥0, while$\overset{}{\;V}\left(t\right)\;\leq 0$; it can be concluded that all the signals in the closed-loop system are bounded, such as the sliding mode variable *s*_*i*_, the joint position tracking error *e*_*i*_ and the estimation error ${\tilde{\Theta }}_{i}$ of the adaptive law. And then we used barbarat's theorem to know that$\overset{}{\;V}\left(t\right)$ asymptotically tends to 0, and then, when *t* → ∞, *s*_*i*_ → 0 and thenė_*i*_ → 0.

Prove (2): from (1), we know the Lyapunov candidate function


(17) $$\begin{array}{l} V_{1}\;=\;\underset{j\;=\;m,s}{\sum }\;\frac{1}{2}S_{j}^{T}S_{j} \end{array}$$

In the same way, it is deduced that:


(18) $$\begin{array}{l} V_{1}\;\leq \;\underset{j\;=\;m,s}{\sum }\;-s_{j}^{T}\bar{K_{j}}s_{j}\;-\;s_{j}^{T}\bar{B_{j}}sig{\left(s_{j}\right)}^{\rho_{j}} \end{array}$$

Therefore, we can get:


(19) $$\begin{array}{l} V_{1}\leq \;-\;S^{T}{\text{Ψ}}_{1}S\;-\;S^{T}{\text{Ψ}}_{2}sig{\left(S\right)}^{\rho_{j}} \end{array}$$

Among which, $sig{\left(S\right)}^{\rho }\;=\;{\left[{\left(sig{\left(s_{m}\right)}^{\rho_{m}}\right)}^{T},\;{\left(sig{\left(s_{S}\right)}^{\rho_{S}}\right)}^{T}\right]}^{T}$.

Then, we can deduce that the convergence time satisfies:


(20) $$\begin{array}{l} T\;\leq \;\frac{1}{\bar{{\text{Ψ}}_{1}}\left(1\;-\;\rho \right)}\;\ln\;\frac{2\bar{{\text{Ψ}}_{1}}V_{1}^{\left(1\;-\;\rho \right)/2}\left(s\left(0\right)\right)\;+\;2^{\left(1\;-\;\rho \right)/2}\bar{{\text{Ψ}}_{2}}}{2^{\left(1-\rho \right)/2}\bar{{\text{Ψ}}_{2}}} \end{array}$$

To sum up, we can prove that the joint position tracking error of the master robot and the slave robot in the closed-loop teleoperation system with time-delay force feedback based on the continuous adaptive terminal sliding mode bilateral controller in this chapter can converge to 0 in finite time, and all the signals of the closed-loop system are bounded, which can not only ensure the stability of the system but also improve the tracking performance of the system.

## Experiments

Simulink is used for simulation verification (Wang et al., 2021), and the S-function is used to establish the system model (Li et al., 2021), and then the closed-loop control system of time-delay force feedback teleoperation system with finite time convergence is built as shown in Figure 1. Compared with the traditional PD (proportional and derivative) control method, the simulation results are analyzed.


(21) $$\begin{array}{l} M_{q_{i}}\left(q_{i}\right)\;=\;\left[\begin{array}{cc} m_{i1}l_{i1}^{2}\;+\;m_{i2}l_{i1}^{2}\;+\;m_{i2}l_{i2}^{2}\;+\;2m_{i2}l_{i1}l_{i2}\cos\left(q_{i2}\right) & m_{i2}l_{i2}^{2}\;+\;m_{i2}l_{i1}l_{i2}\cos\left(q_{i2}\right)\\ m_{i2}l_{i2}^{2}+m_{i2}l_{i1}l_{i2}\cos\left(q_{i2}\right) & m_{i2}l_{i2}^{2} \end{array}\right] \end{array}$$

(22) $$C_{q_{i}}(q_{i},{\dot{q}}_{i})\;=\;\left[\begin{array}{cc} -m_{i2}l_{i1}l_{i2}{\dot{q}}_{i2}cos\;(q_{i2}) & -m_{i2}l_{i1}l_{i2}({\dot{q}}_{i1}+{\dot{q}}_{i2})\sin(q_{i2}\\ m_{i2}l_{i1}l_{i2}{\dot{q}}_{i1}\sin(q_{i2}) & 0 \end{array}\right]$$

(23) $$\begin{array}{l} G_{q_{i}}\left(q_{i}\right)\;=\left[\begin{array}{c} \left(m_{i1}l_{i2}+m_{i2}l_{i1}\right)g\cos\left(q_{i1}\right)+m_{i2}l_{i2}g\cos\left(q_{i1}+q_{i2}\right)\\ m_{i2}l_{i2}g\cos\left(q_{i1}+q_{i2}\right) \end{array}\right] \end{array}$$

In this paper, the master robot and the slave robot in the teleoperation system adopt the 2-DOF, 2-link, rotary joint robot. For the sake of simplicity and generality, the moment of inertia of the rod is ignored. The mathematical models of joint space dynamics are as follows:

In addition, the external interference of the master robot and the slave robot in the system is also set as $f_{i}\left(q_{i},{\overset{∙}{q}}_{i}\right)={\left[\begin{array}{cc} 0.1q_{i1}{\overset{∙}{q}}_{i1}\sin\left(t\right) & 0.1q_{i2}{\overset{∙}{q}}_{i2}\sin\left(t\right) \end{array}\right]}^{T}$, and the internal friction of the master robot and the slave robot is $f_{cm}\left({\overset{∙}{q}}_{m}\right)={\left[\begin{array}{cc} f_{d1}{\overset{∙}{q}}_{m1}+k_{1}\mathrm{sign}\left({\overset{{}^{\circ}}{q}}_{m1}\right) & f_{d2}{\overset{∙}{q}}_{m2}+k_{2}\mathrm{sign}\left({\overset{{}^{\circ}}{q}}_{m2}\right) \end{array}\right]}^{T},$respectively, and $f_{cs}\left({\overset{∙}{q}}_{s}\right)={\left[f_{d3}{\overset{∙}{q}}_{s1}+k_{3}sign\left({\overset{∙}{q}}_{s1}\right)f_{d4}{\overset{∙}{q}}_{s2}+k_{4}sign\left({\overset{∙}{q}}_{s2}\right)\right]}^{T}$, where *f*_*d*1_, *f*_*d*2_, *k*_1_, *k*_2_ are constants, and *i* = *m, s*.

At the same time, the external force from the operator is selected as $f_{h}^{*}\;=\;{\left[25\left(1\;-\;\cos\left(\pi t\right)\right)0\right]}^{T}$, and the external force from the interaction between the robot and the environment is selected as $f_{e}^{*}\;=\;{\left[\begin{array}{cc} 0 & 0 \end{array}\right]}^{T}$.

In the process of building a closed-loop teleoperation system, the mechanical constant parameters related to the dynamics of the master robot, the slave robot, the operator, and the environment are shown in Table 1.

**Table 1:** Master-slave robot parameters and operator and environment parameters.

**m_m1_**	**l_m1_**	**m_m2_**	**l_m2_**	**m_s1_**	**l_s1_**	**m_s2_**	**l_s2_**
0.5 kg	0.6 m	0.5 kg	0.4 m	0.5 kg	0.6 m	0.5 kg	0.4 m
g							
9.81 *m*/*s*^2^	1	2	3	3	3	2	4
*k*_4_	*M*_*h*_	*B*_*h*_	*K*_*h*_	*M*_*e*_	*B*_*e*_	*K*_*e*_	
6	0.2 kg	50 *Ns*/*m*	1,000 *N*/*m*	0.1 kg	20 *Ns*/*m*	1,000 *N*/*m*	

*$sig{\left(\xi \right)}^{\alpha }\;=\;{\left[{\left|\xi_{1}\right|}^{\alpha_{1}}sign\left(\xi_{1}\right),{\left|\xi_{2}\right|}^{\alpha_{2}}sign\left(\xi_{2}\right),\cdots \;,{\left|\xi_{n}\right|}^{\alpha_{n}}sign\left(\xi_{n}\right)\right]}^{T}$ $\xi ={\left[\xi_{1},\xi_{2},\cdots \;,\xi_{n}\right]}^{T}\in R^{n}$ α_1_, α_2_, ⋯ , α_n_ > 0*.

In the simulation, it is assumed that the uncertain part of the master robot's dynamic model is Δ*M*_*m*_ = 0.3*sin*(2*t*)*M*_*om*_, Δ*C*_*m*_ = 0.2*sin*(3*t*)*C*_*om*_, Δ*G*_*m*_ = 0.1*sin*(4*t*)*G*_*om*_ and that of the slave robot's dynamic model is $q_{m}\left(0\right)={\left[\begin{array}{c} 0.4pi\&0.2pi \end{array}\right]}^{T},\;q_{s}\left(0\right)={\left[\begin{array}{c} 0.1pi\&0.05pi \end{array}\right]}^{T}$. Set the initial position of the master robot and the slave robot. The time delay of forward and reverse communication channels of teleoperation system is *T*_*m*_ = *T*_*s*_ = 0.6*s*.

In the simulation teleoperation system, the master robot and the slave robot controller adopt Formula (9) and Formula (10). After repeated debugging, the controller parameters in the remote operation system are *K*_*m*_ = *K*_*s*_ = *diag*(3, 3), *B*_*m*_ = *B*_*s*_ = *diag*(3, 3),α_*m*_ = α_*s*_ = *diag*(1, 1),β_*m*_ = β_*s*_ = *diag*(1, 1), ε_*m*_ = ε_*s*_ = *diag*(3, 3), γ_*m*_ = γ_*s*_ = *diag*(1.5, 1.5),ρ_*m*_ = ρ_*s*_ = *diag*(1/3, 1/3). The adaptive law is equation. After repeated debugging, its parameters are Λ_*m*1_ = Λ_*s*1_ = *diag*(2, 2), Λ_*m*1_ = Λ_*s*1_ = *diag*(0.5, 0.5).

In order to further observe whether the teleoperation system can keep stable if the external force changes due to the interaction between the robot and the environment, in the simulation, we reset $f_{e}^{*}={\left[\begin{array}{c} 0\&0 \end{array}\right]}^{T}$as $f_{e}^{*}={\left[\begin{array}{c} 20\&20 \end{array}\right]}^{T}$at runtime*t* = 4*s*. Meanwhile, we reset *K*_*e*_ = 1, 000 as *K*_*e*_ = 1, 100.

In order to explain the advantages of the continuous adaptive terminal sliding mode bilateral controller objectively, comparative experiment is carried out. In the simulation, after repeated debugging, the parameters *L*_*m*_, *L*_*s*_, *N*_*m*_, *N*_*s*_ in the controller are *L*_*m*_ = *L*_*s*_ = *diag*(100, 100), *N*_*m*_ = *N*_*s*_ = *diag*(100, 100), respectively.

The bilateral PD controller proposed in Reference 16 is chosen for comparative simulation. The expression of the controller is as follows:


(24) $$\begin{array}{l} \tau_{m}\;=\;-L_{m}\left(q_{m}\left(t\right)\;-\;q_{s}\left(t-T_{s}\right)\right)\;-\;N_{m}{\overset{∙}{q}}_{m}\;+\;G_{m} \end{array}$$

(25) $$\begin{array}{l} \tau_{s}\;=\;-L_{s}\left(q_{s}\left(t\right)\;-\;q_{m}\left(t-T_{m}\right)\right)\;-\;N_{s}{\overset{∙}{q}}_{s}\;+\;G_{s} \end{array}$$

## Results

**Figure 2:**
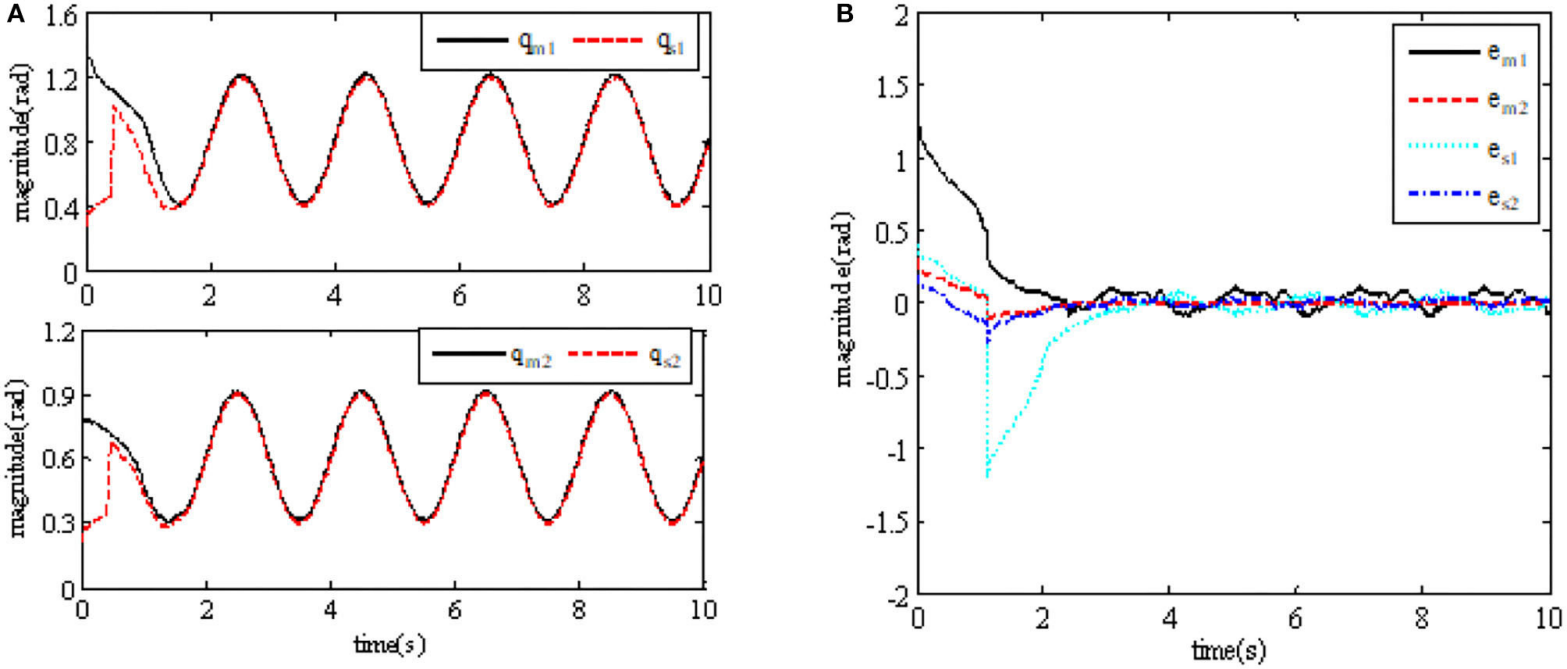
Tracking performance between master and slave robots. **(A)** Tracking of master and slave robots' joints; **(B)** position tracking error of master and slave robots' joints.

**Figure 3:**
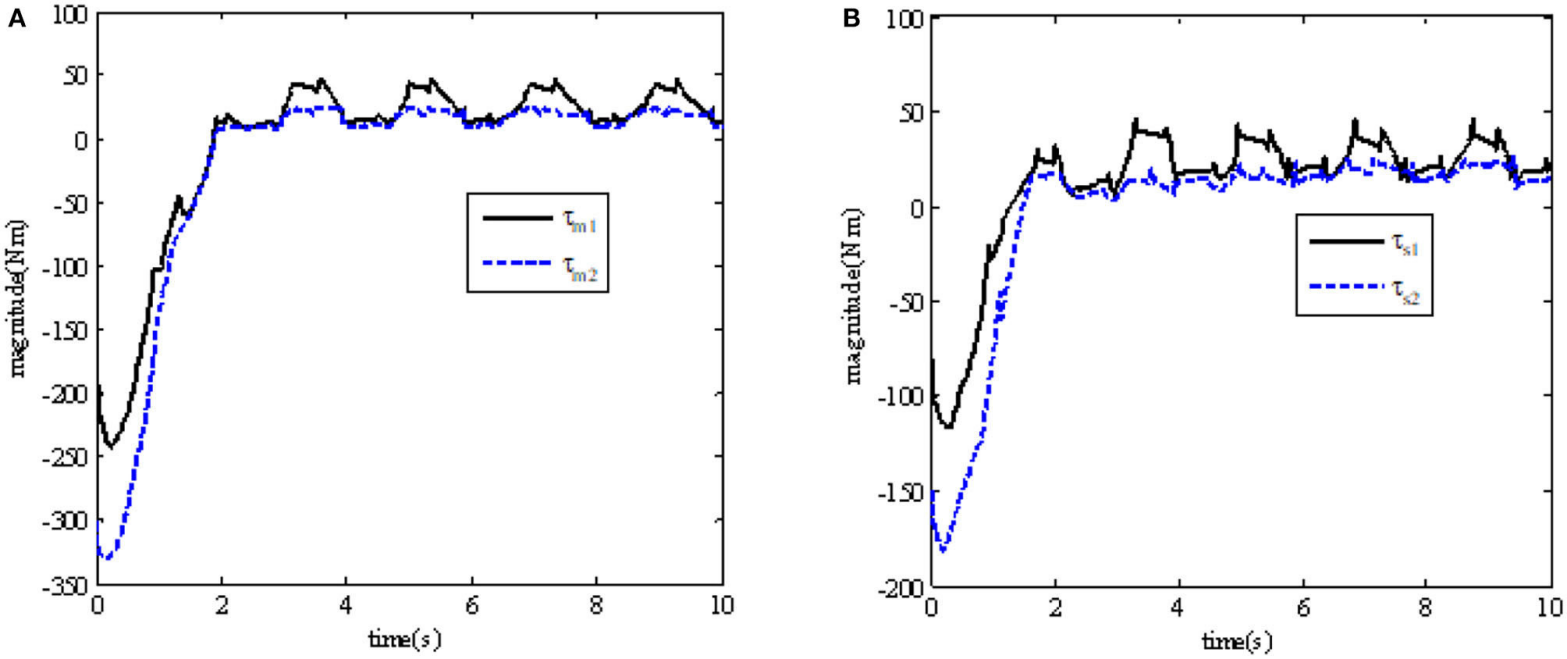
Input torque τ_*m*_ and τ_*s*_ of robot joints. **(A)** The master robot joint input torqueτ_*m*_; **(B)** the slave robot joint input torque τ_*s*_.

**Figure 4:**
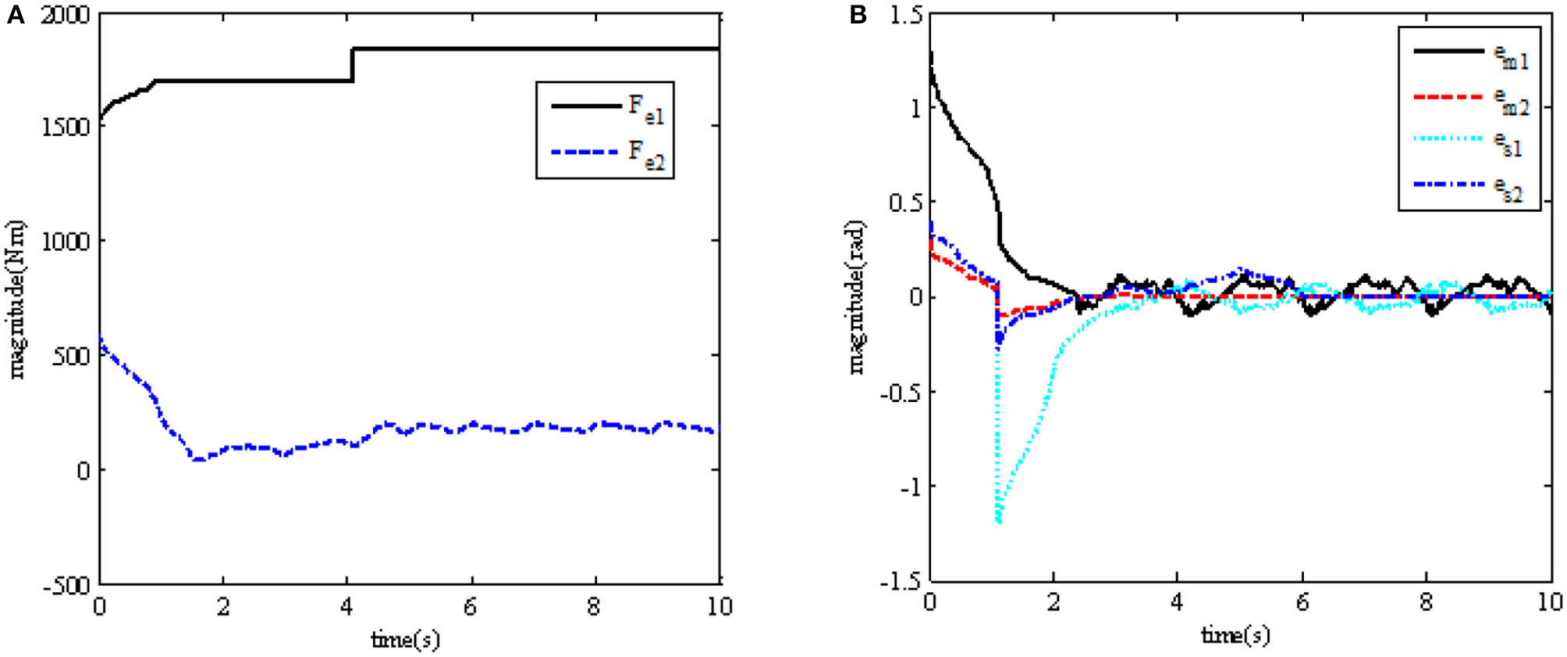
Simulation results after the change of external force *F*_*e*_. **(A)** From the interaction, *F*_*e*_ between the robot and the environment; **(B)** position tracking error of master and slave robots' joints.

In order to illustrate the effectiveness of using the continuous adaptive terminal sliding mode control bilateral controller in the closed-loop teleoperation system with time-delay force feedback, the simulation results are shown in Figures 2, 3. Figure 2 shows the tracking performance between the master and slave robots of the teleoperation system. Figure 3 shows the input torque signals of Joint 1 and Joint 2 of the master robot and the slave robot in the system,

In order to further observe whether the teleoperation system can continue to maintain stability when the external force changes due to the interaction between the robot and the environment, the simulation results of position tracking error and the environmental force change are shown in (a) and (b) in Figure 4.

**Figure 5:**
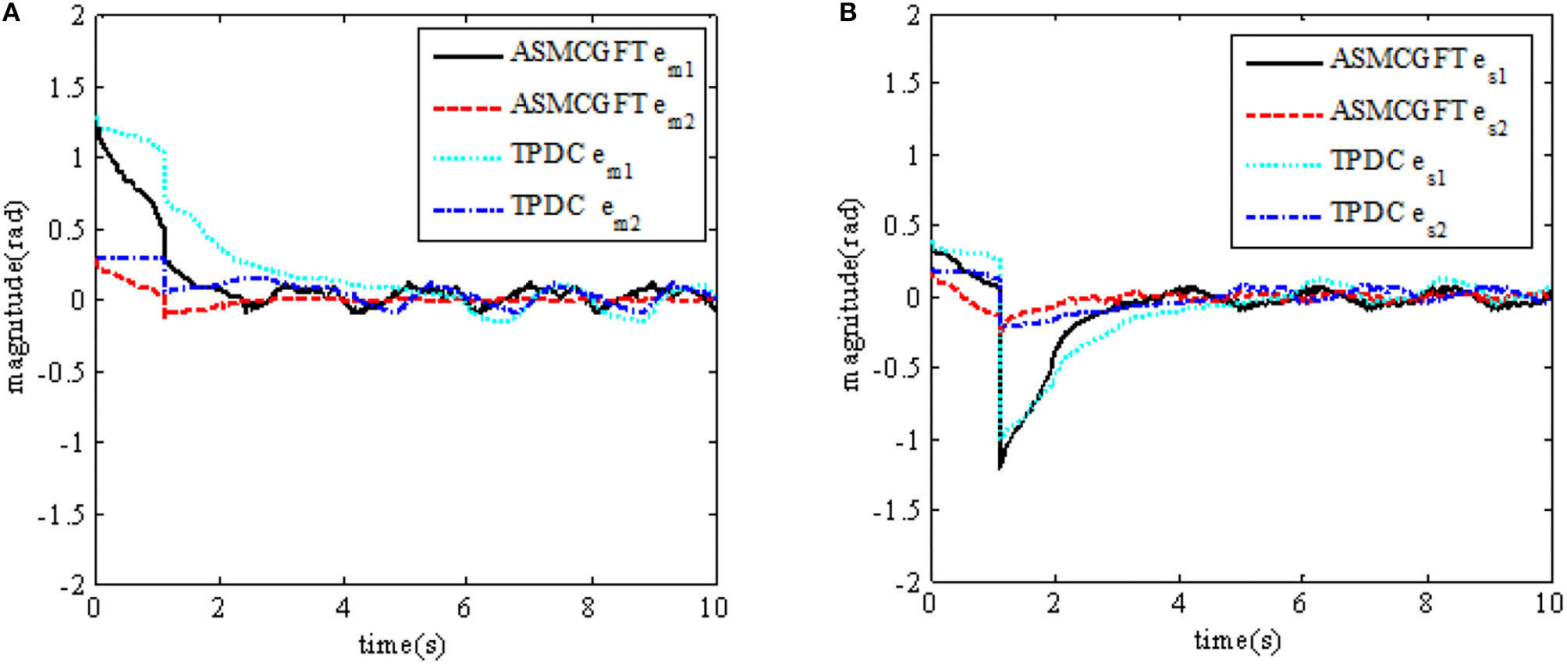
Comparison of tracking error between master and slave robots. **(A)** Comparison of the master robot's joint position tracking error **(B)** Comparison of the slave robot's joint position tracking error.

**Figure 6:**
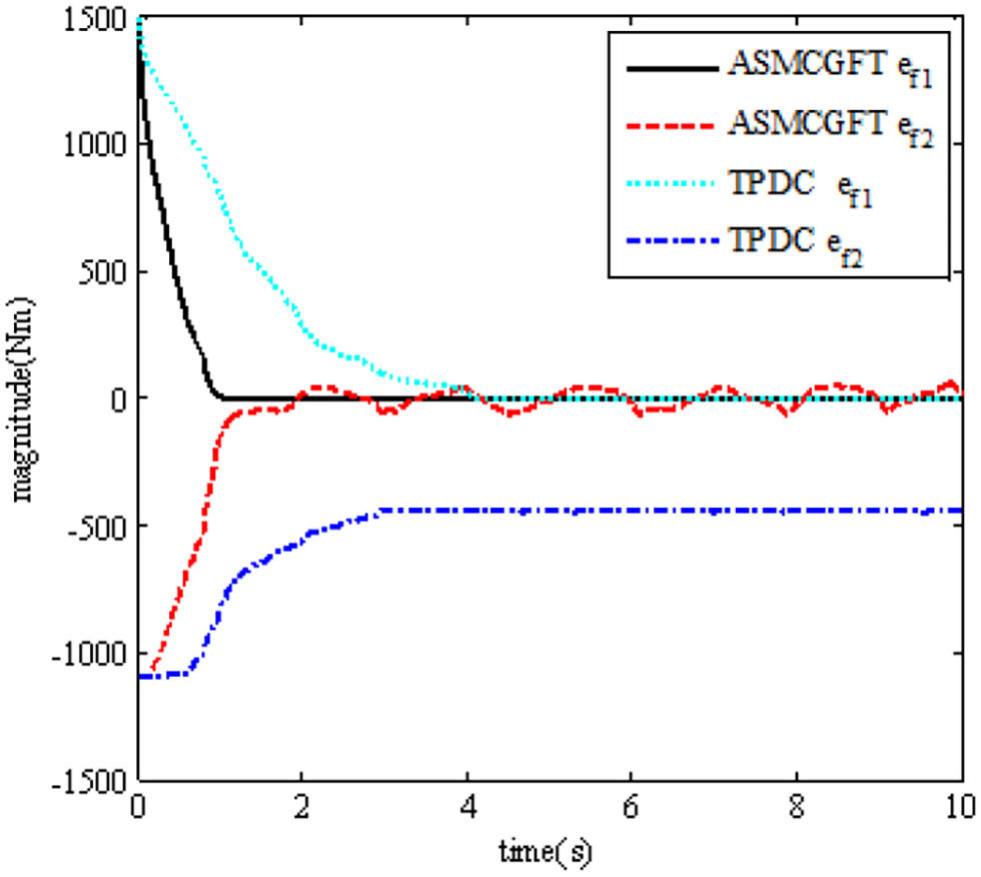
Comparison of master-slave robot contact torque error.

In order to explain the advantages of the continuous adaptive terminal sliding mode bilateral controller objectively, comparative experiments were carried out, and the experiment results are shown in Figures 5, 6. Figures 5, 6 show the comparison of angular position tracking errors of the Joint 1 and Joint 2 of the master robot and the slave robot under the control method in this paper and the traditional PD control method, respectively. “ATSMCGFT” refers to “adaptive terminal sliding mode bilateral controller with guaranteed continuous finite time”; “TPDC” refers to “traditional proportional and derivative bilateral controller.”

In addition, Figure 6 shows the comparison diagram of the contact force tracking error between the master robot and the slave robot.

## Discussion

The controller of the master robot and the slave robot is designed based on the non-singular terminal sliding mode control method, and the neural network adaptive method is also incorporated into the controller to approximate the uncertainty of the teleoperation system model so as to eliminate the influence of the system model uncertainty on the system stability. Based on Lyapunov stability theory and terminal sliding mode control theory, the stability of the teleoperation system with time-delay force feedback and the tracking error of the master robot and the slave robot can converge to 0 in limited time. Based on the theory of the terminal sliding mode control, the non-linear sliding mode variable is defined, and the appropriate controller algorithm is designed to solve the chattering and singularity problems. The ASMCGFT method proposed in the manuscript has a smaller convergence time. The experimental data results show that using the time-delay force feedback teleoperating system of this method, although the joint position tracking error of the master and slave robots can converge to 0 in a limited time, that is, the convergence time of the tracking error has been improved, the average tracking error index is slightly lower. There exists a decrease in error accuracy.

## Conclusion

Through experiments, we can see that the robot can track the movement of the upper master robot in 2 s, and from the simulation experiment results that the position tracking error of the master robot and the slave robot of the teleoperation system in this paper can quickly converge to zero, and the system is globally stable and has good instantaneous characteristics.

Besides, we also can observe that the input torque of each joint of the master and slave robot under the control method designed in this paper is bounded. At the same time, we can also see that the slave robot can track the upper master robot in 2 s. The experimental results show the control method designed in this paper has good performance.

It can be seen from the results that, when *t* = 4 *s*, after the environmental force becomes larger, the tracking error of the teleoperation system can also be adjusted to the area near 0 in a limited time, while maintaining the stability of the system.

It also can be seen from the results that the convergence time of the position tracking error *e*_*m*_ of the master robot under the control method in this paper is about ${\left[\begin{array}{cc} 2 & 2 \end{array}\right]}^{T}$, and that of the position tracking error *e*_*s*_ of the slave robot is about ${\left[\begin{array}{cc} 2 & 2.5 \end{array}\right]}^{T}$. While the convergence time of the master robot position tracking error *e*_*m*_ under the traditional PD control method is about ${\left[\begin{array}{cc} 6 & 5 \end{array}\right]}^{T}$, and the convergence time of the slave robot position tracking error *e*_*s*_ is about ${\left[\begin{array}{cc} 4.5 & 5.2 \end{array}\right]}^{T}$.

To sum up, from the comparison of experimental results, we can observe that the control method in this paper has better performance; the tracking error of its position and contact force can converge to near 0 in a short time; at the same time, it has good performance of force feedback-tracking control.

## Data Availability Statement

The raw data supporting the conclusions of this article will be made available by the authors, without undue reservation.

## Author Contributions

SL, BY, WZ, and LY contributed to the design of this work. JW, JT, and XZ contributed to the writing of the manuscript. JW and JT designed the model and implemented it in the framework, together with WZ and LY revised the manuscript. All authors contributed to the article and approved the submitted version.

## Funding

This research was funded by the Sichuan Science and Technology Program, Grant No. 2021YFQ0003.

## Conflict of Interest

The authors declare that the research was conducted in the absence of any commercial or financial relationships that could be construed as a potential conflict of interest.

## Publisher's Note

All claims expressed in this article are solely those of the authors and do not necessarily represent those of their affiliated organizations, or those of the publisher, the editors and the reviewers. Any product that may be evaluated in this article, or claim that may be made by its manufacturer, is not guaranteed or endorsed by the publisher.
